# ESR1 fusions and therapeutic resistance in metastatic breast cancer

**DOI:** 10.3389/fonc.2022.1037531

**Published:** 2023-01-04

**Authors:** Zsuzsanna Nagy, Rinath Jeselsohn

**Affiliations:** ^1^ Center for Functional Cancer Epigenetics, Dana Farber Cancer Institute, Harvard Medical School, Boston, MA, United States; ^2^ Department of Medical Oncology, Dana-Farber Cancer Institute, Boston, MA, United States; ^3^ Department of Medicine, Harvard Medical School, Boston, MA, United States; ^4^ Susan F. Smith Center for Women’s Cancers, Dana-Farber Cancer Institute, Harvard Medical School, Boston, MA, United States

**Keywords:** breast cancer, estrogen receptor, ESR1 fusion, endocrine therapy resistance, SERD

## Abstract

Breast cancer is the most frequent female malignant tumor, and the leading cause of cancer death in women worldwide. The most common subtype of breast cancer is hormone receptor positive that expresses the estrogen receptor (ER). Targeting ER with endocrine therapy (ET) is the current standard of care for ER positive (ER+) breast cancer, reducing mortality by up to 40% in early- stage disease. However, resistance to ET represents a major clinical challenge for ER+ breast cancer patients leading to disease recurrence or progression of metastatic disease. Salient drivers of ET resistance are missense mutations in the ER gene (*ESR1*) leading to constitutive transcriptional activity and reduced ET sensitivity. These mutations are particularly prominent and deleterious in metastatic breast cancer (MBC). In addition to activating *ESR1* point mutations, emerging evidence imposes that chromosomal translocation involving the *ESR1* gene can also drive ET resistance through the formation of chimeric transcription factors with constitutive transcriptional activity. Although these *ESR1* gene fusions are relatively rare, they are enriched in ET resistant metastatic disease. This review discusses the characteristics of ER fusion proteins and their association with clinical outcomes in more aggressive and metastatic breast cancer. The structure and classification of ER fusion proteins based on function and clinical significance are also addressed. Finally, this review summarizes the metastatic phenotypes exhibited by the ER fusion proteins and their role in intrinsic ET resistance.

## Introduction

Despite significant advances in breast cancer screening and treatment, mortality rates remain high with nearly 2.3 million new cases diagnosed and more than 650 000 patients dying each year worldwide according to the World Health Organization (1). The most common breast cancer subtype is hormone receptor positive, expressing the ER and/or progesterone receptor, accounting for approximately 75% of breast cancers (2). ER is a nuclear transcription factor that drives breast cancer development and growth. ER is comprised of four domains (3), an N-terminal activation function-1 (AF-1), a central DNA binding domain followed by a hinge region and the C-terminal ligand binding domain (LBD) that contains the ligand- dependent activation function (AF-2). Following estrogen binding, ER dimerizes and translocate to the nucleus where it binds to DNA at estrogen response elements (ERE) to regulate the transcription of multiple genes involved in tumor progression (3). ER functions as part of a transcriptional complex including (1) other transcription factors, such as Activator Protein 1 (AP1), Transcription Factor SP1 (SP1), Nuclear Factor-κB (NF-κB) and E2F Transcription Factor 1 (E2F1) (4–8); (2) co-factors that regulate chromatin structure, such as Nuclear Receptor Coactivator 1 (SRC-1), Nuclear Receptor Coactivator 2 (TIF2), Glutamate Receptor Interacting Protein 1 (GRIP-1), Amplified in Breast Cancer 1 (AIB1), CREB binding protein (CBP), p300 and the p300/CBP-associated factor (pCAF) (9–16); (3) pioneer factors that modulate ER binding to chromatin, such as Forkhead Box A1 (FOXA1), GATA Binding Protein 3 (GATA3), Pre-B-cell Leukemia Transcription Factor 1 (PBX1), and transducin-like enhancer protein 1 (TLE1) (17–23). ET is the mainstay treatment in ER+ breast cancer (24), and these treatment options include selective estrogen receptor modulators (SERMs), aromatase inhibitors (AI), selective estrogen receptor degraders (SERDs), and selective estrogen receptor covalent antagonists (SERCAs). SERMs, such as tamoxifen antagonize ER by reducing co-factor binding (25). AIs block the conversion of testosterone to estrogen and SERDs, such as fulvestrant, competitively bind ER and lead to proteasomal degradation (25). SERCA H3B-5942 inactivates ER by targeting Cys530 to enforce a distinct antagonist conformation (26, 27). Combination of ET with inhibitors for cyclin dependent kinase 4/6 (CDK4/6), mammalian target of rapamycin (mTOR), or phosphatidylinositol-4,5- biphosphate 3-kinase catalytic subunit alpha (PIK3CA) is integral part of the treatment in metastatic ER+ breast cancer and represent major improvements in progression free survival (28).

While ET reduces mortality by up to 40% in early-stage disease and highly effective in controlling metastatic disease, therapeutic resistance remains a momentous clinical issue (29, 30). At most, 20% of resistant cases lose ER expression (31) and in many patients the ER transcriptional axis remains active, however, in an altered fashion. To date, multiple mechanisms of acquired resistance to ET have been investigated and identified. These include (1) altered expression of transcription factors and co-regulatory proteins (e.g. SP1, AP1, NF-κB, SRC-1, AIB1, FOXA1) (18, 32–37), (2) modification of ER by miRNAs (e.g. miR-148, miR-152 and miR-221/222) (38–41), (3) increased crosstalk between ER/HER2/SRC3 (42), (4) amplification of tyrosine kinase receptors (e.g. fibroblast growth factor receptor 1 and 2 or insulin-like growth factor receptor 1) (43–46), (5) aberrant expression of cell cycle proteins (e.g. c-Myc, p21 and p27) (47–52) and (6) immune system-dependent resistance regulated *via* the NF-κB pathway (53) or chemokines activated PI3K/Akt/mTOR signaling (54, 55). Additional mechanism of ET resistance is the acquisition of somatic mutations in *ESR1* that are present in up to 50% of MBC patients (56–61). Widely studied examples are point mutations in the LBD (Y537S and D538G) that confer ER constitutive activity and exhibit decreased ET sensitivity (57, 59).

Studies have shown that chimeric proteins are powerful drivers of cancer with tremendous clinical impact (62). Larotrectinib, the first pan-cancer drug against the NTRK gene fusions demonstrated rapid responses in both adult and pediatric cancer patients (63–68). Driven by deep transcriptomic sequencing studies, several pathological gene fusions have been identified in aggressive (luminal B, basal like, or endocrine resistant breast cancer) breast cancers (69–72). These include fusion proteins associated with ER such as ESR1-CCDC170 (73–75), and ESR1-YAP1 (76) and non ER related fusions such as CTNNBL1-RAF1, ACTL6A-PIK3CA, S6KCI-AKT3 (71), SEC16A-NOTCH1 (77), SEC22B-NOTCH2 (72), and ETV6-NTRK3 (78). A number of these fusions promote tumor growth, and patients expressing these fusion proteins have more rapid disease progression and shorter survival than fusion-negative patients (70, 71, 75, 79). Identifying the full spectrum of the *ESR1* gene fusions and characterizing their role in intrinsic ET resistance is critical for developing novel and effective targeted therapies.

## ESR1 fusions are acquired and enriched in MBC

**Figure 1:**
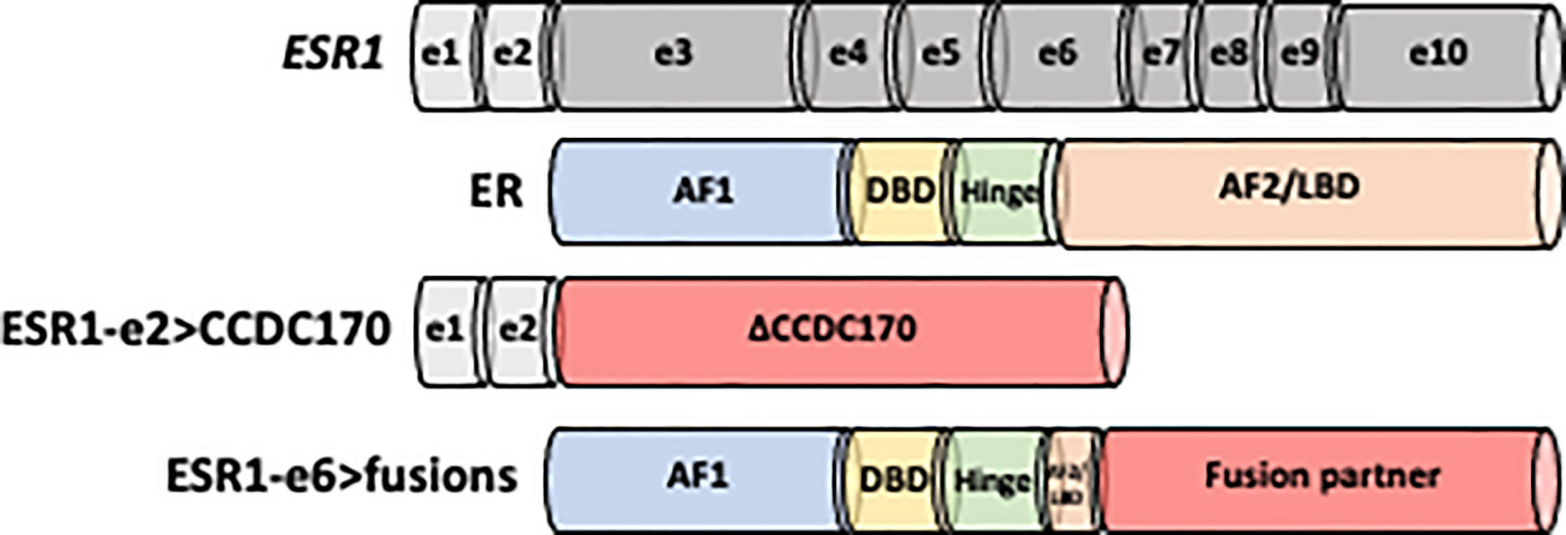
Schematic structure of ESR1-e2>CCDC170 and ESR1-e6>fusion proteins. Non-coding exons (e) 1 and 2 are shown as white boxes, while encoding domains in the ESR1 codon structure are presented in gray. ER is comprised of four domains: N-terminal activation function-1 (AF-1), DNA binding domain (DBD), hinge region and C-terminal ligand binding domain (AF-2/LBD). ESR1-e2>CCDC170 fusion proteins retain the first two non-coding exons of *ESR1* (ESR1-e2) and link to the coiled-coil domain containing 170 (CCDC170) gene generating truncated CCDC170 proteins (ΔCCDC170). ESR1-e6>fusions preserve the first 6 exons of *ESR1* (ESR1-e6), while replace the LBD by a 3’ fusion partner.

RNA-seq analysis conducted by Veeraraghavan and colleagues on 990 primary TCGA breast samples identified the first *ESR1* gene fusion, ESR1-e2>CCDC170 (**Table 1**), in a subset (2.1%) of Luminal B breast tumor samples (75, 82). This fusion is formed by tandem-duplication, it retains the first two non-coding exons of *ESR1* (ESR1-e2) connected to various sequences from the coiled-coil domain containing 170 (CCDC170) gene (**Figure 1**). The promoter trap drives aberrant expression of CCDC170 and produces N-terminal truncated forms of the CCDC170 protein (ΔCCDC170) (75). The authors also provided functional evidence that this fusion promotes more aggressive oncogenic phenotypes in ER+ breast cancer cells, such as increased cell migration, invasion, and reduced tamoxifen sensitivity. Utilizing RNA sequencing, Li and colleagues (76) described the first inter-chromosomal ESR1 fusion in a patient-derived xenograft from ET resistant MBC (Luminal A subtype, skin metastasis). This fusion is formed by a translocation event that brought *ESR1* exons 1 to 6 (ESR1-e6) on chromosome (chr) 6q into the yes associated protein 1 gene *YAP1* locus on chr11q (ESR1- e6>YAP1; **Table 1**), replacing the LBD of ESR1 with the transactivation domain (TAD) sequence from YAP1 (**Figure 1**). Although Li and colleagues conducted limited functional studies, overexpression of ESR1-e6>YAP1 in ER+ breast cancer cells conferred estradiol-independent growth in their study (**Table 1**). Lei and colleagues (80) and Gou and colleagues (81) provided additional mechanistic data for the ESR1-e6>YAP1 fusion and described its functional properties in driving estrogen-independent growth, constitutive expression of ER target genes, and anti-estrogen resistance. Several years later, two additional ESR1 fusions, ESR1-e2 fusion with the acidic residue methyltransferase 1 gene, C6orf211/ARMT1 (ESR1-e2>C6orf211/ARMT1) and ESR1-e6 fusion with a-kinase anchoring protein 12 gene, AKAP12 (ESR1-e6>AKAP12) were identified in AI resistant breast cancer by Giltnane and colleagues (**Table 1**) (82) with no functional data available (**Table 1**). Using whole genome sequencing, Robinson and colleagues (83) identified three additional ESR1 fusions (**Table 1**) including fusionsaryl hydrocarbon receptor nuclear translator 2 gene, ARNT2 (ESR1-e6>ARNT2-e18); protein-L-isoaspartate O-methyltransferase gene, PCMT1 (ESR1-e6>PCMT1); AT-rich interaction domain 1B gene, ARID1B (ESR1-e6>ARID1B), but the functional properties of these fusions were investigated only later on by Gou and colleagues (81). Hartmaier and colleagues also described the expression of ESR1-e6>AKAP12 in ER+ MBC and identified several novel ESR1 fusions (84). The authors also established that ESR1 fusion proteins are enriched in ER+ MBC (**Table 1**) and contribute to ET resistance. Hartmaier and colleagues described eight novel ESR1 fusion proteins (**Table 1**), all with junctions between ESR1 exon 6 and 7. ESR1-e6>fusions included DAB adaptor protein 2 gene, DAB2 (ESR1-e6>DAB2); glycogenin-1 gene, GYG (ESR1-e6>GYG1); SRY-box transcription factor 9 gene, SOX9 (ESR1-e6>SOX9); pleckstrin homology and RhoGEF domain containing G1, PLEKHG1 (ESR1-e6>PLEKHG1); trafficking from ER to Golgi regulator, TFG (ESR1-e6>TFG); and sodium/potassium transporting ATPase interacting 2, NKAIN2 (ESR1-e6>NKAIN2). ESR1-e7>fusions included mitochondrial isozyme of C1-tetrahydrofolate (THF) synthase, MTHFD1L (ESR1-e7>MTHFD1L) and cyclin dependent kinase 13, CDK13 (ESR1-e7>CDK13). The authors emphasized that many genetic rearrangement events are not expressed or translated into functional protein products, therefore they utilized an array of techniques (DNA and/or RNA sequencing, PCR and immunoblot) to investigate fusion protein expression (**Table 1**). As example, ESR1-e6>DAB2 and ESR1-e6>GYG1 were detected by DNA and/or RNA sequencing, and immunoblot, and *in vitro* studies established that these fusions were stable and active. The authors were able to detect ESR1-e6>SOX9, ESR1-e7>MTHFD1L, ESR1-e6>PLEKHG1, ESR1-e6>NKAIN2, ESR1-e6>AKAP12, and ESR1-e7>CDK13 only by DNA sequencing with low confidence in producing fusion transcripts. Utilizing anchored multiplex PCR, Matissek and colleagues (71) identified an additional ESR1 fusion protein (**Table 1**) with junction between ESR1 exon 4 and 3’ fusion partner cytochrome C oxidase assembly factor 5, COA5 (ESR1-e4>COA5). The role of this fusion in MBC and ET resistance is currently unknown. Lei and colleagues (80) conducted a comprehensive study and identified several novel *ESR1* fusions (**Table 1**). The protocadherin 11 X-linked fusion (ESR1-e6>PCDH11X**)** was identified from a male patient with ER+ MBC. Inter-chromosomal *ESR1* translocations included the nucleolar protein 2 homolog gene, NOP2 (ESR1-e6>NOP2), and aldo-keto reductase family 1 member D1 (ESR1-e6>AKR1D1). Fusion with DNA polymerase eta gene, POLH (ESR1-e7>POLH) was formed by intra-chromosomal translocation. In this study, the authors also identified the ESR1-e4>CCDC170 and ESR1-e5>CCDC170 fusions. Lei and colleagues conducted functional studies and provided further evidence that ESR1-e6>YAP1 and ESR1-e6>PCDH11X, identified from ER+ MBC, encoded stable and functional fusion proteins and promoted estrogen-independent growth, induced cellular motility, constitutive expression of ER target genes, and anti-estrogen resistance. Seven additional ESR1-e6>fusions (**Table 1**) were identified by Priestley and colleagues in ER+ MBC, including aqryl hydrocarbon receptor nuclear translocator 2, ARNT2 (ESR1-e6>ARNT2); LIM domain containing preferred translocation partner in lipoma, LPP (ESR1-e6>LPP); nuclear receptor coactivator 1, NCOA1 (ESR1-e6>NCOA1); transcription factor 12, TFC12 (ESR1-e6>TCF12); clathrin interactor 1, CLINT1 (ESR1-e6>CLINT1); glutamate receptor interacting protein 1, GRIP1 (ESR1-e6>GRIP1) and trinucleotide repeat containing adaptor 6B, TNRC6B (ESR1-e6>TNRC6B). Functional characterization of these fusions were investigated by Gou and colleagues (81, 85) (**Table 1**). Except ESR1-e6>TCF12, all ESR1-e6>fusions promoted estrogen-independent growth. It is noteworthy that up to date, only few ESR1 fusions (ESR1-e2>CCDC170, ESR1-e4>CCDC170, ESR1-e5>CCDC170, ESR1-e6>NOP2, ESR1-e6>AKR1D1, ESR1-e6>POLH) were detected in primary breast cancer samples (**Table 1**).

**Table 1:** Summary of ESR1 gene fusions identified in breast cancers.

ESR1 fusion	Study cohorts and incidence	Clinical characteristics	Detection methods	Reference for detection	ESR1 break point exon	Frame	Functional fusion expression	Function	Reference for function
ESR1-CCDC170	cohort of 990 TCGA breast samples; 21 of 990 tumors (2.1%)	primary; luminal B subtype	RNA sequencing; PCR	(75)	exon 2	5’UTR-CDS	stable expression; produce truncated CCDC170 protein (rather than a chimeric protein)	increased cell migration and anchorage independent growth; increased colony-formation; reduced tamoxifen sensitivity	(75)
ESR1-YAP1	cohort of 22 patient-derived xenografts; 1 of 22 tumors	endocrine therapy resistant, metastatic ER+	RNA sequencing	(76)	exon 6	in-frame	stable expression and active fusion protein	estrogen independent growth; fulvestrant resistant growth; constitutive expression of ER and EMT-like transcriptional programs; increased cell motility and development of lung metastasis	(76) (80) (81)
ESR1-c6orf211/ARMT1	cohort of 15; 2 of 15 tumors	early stage (stage I-III) and non-metastatic ER+	RNA sequencing; Nano-string; PCR	(82)	exon 2	5’UTR-CDS	unknown	unknown	
ESR1-AKAP12	cohort of 15; 1 of 15 tumors	early stage (stage I-III) and non-metastatic ER+	RNA sequencing; Nano-string; PCR	(82)	exon 6	in-frame	unknown	unknown	
ESR1-ARNT2-e18	cohort of 91 breast cancer patients (MET500 cohort); 1 of 91	metastatic ER+	whole exome sequencing	(83)	exon 6	in-frame	stable expression and active fusion protein	estrogen independent growth; fulvestrant resistant growth; constitutive expression of ER and EMT-like transcriptional programs; increased cell motility	(81)
ESR1-PCMT1	cohort of 91 breast cancer patients (MET500 cohort); 1 of 91	metastatic ER+	whole exome sequencing	(83)	exon 6	in-frame	stable expression and inactive fusion protein	no role in estrogen independent and ET resistant growth	(81)
ESR1-ARID1B	cohort of 91 breast cancer patients (MET500 cohort); 1 of 91	metastatic ER+	whole exome sequencing	(83)	exon 6	in-frame	stable expression and inactive fusion protein	no role in estrogen independent and ET resistant growth	(81)
ESR1-DAB2	cohort of 6 patient-matched breast cancer samples (University of Pittsburgh Health Science Tissue Bank); 1 of 6 tumors	supraclavicular lymph node metastasis; ER+	whole-genome sequencing RNA sequencing PCR immunoblot	(84)	exon 6	in-frame	stable expression and active context-dependent fusion protein	estrogen independent growth in MCF7 but not T47D cells	(81)
ESR1-GYG1	cohort of 6 patient-matched breast cancer samples (University of Pittsburgh Health Science Tissue Bank); 1 of 6 tumors	bone metastasis; ER+	whole-genome sequencing RNA sequencing PCR immunoblot	(84)	exon 6	in-frame	stable expression and inactive fusion protein	no role in estrogen independent and ET resistant growth	(81)
ESR1-SOX9	cohort of 9542 breast tumors (5216 from metastatic disease) from patients with advanced breast cancer (Foundation Medicine); 1 of 9542 (0.01%)	metastatic ER+ (solid tumor; liver metastasis)	comprehensive genomic profiling	(84)	exon 6	in-frame	stable expression and active fusion protein	estrogen independent growth; fulvestrant resistant growth; constitutive expression of ER and EMT-like transcriptional programs; increased cell motility	(84) (81)
ESR1-MTHFD1L	cohort of 9542 breast tumors (5216 from metastatic disease) from patients with advanced breast cancer (Foundation Medicine); 1 of 9542 (0.01%)	late-stage, endocrine-refractory (solid tumor; local reoccurrence)	comprehensive genomic profiling	(84)	exon 7	in-frame	unknown	unknown	
ESR1-PLEKHG1	cohort of 9542 breast tumors (5216 from metastatic disease) from patients with advanced breast cancer (Foundation Medicine); 1 of 9542 (0.01%)	late-stage, endocrine-refractory, metastatic (solid tumor; liver metastasis)	comprehensive genomic profiling	(84)	exon 6	in-frame	unknown	unknown	
ESR1-TFG	cohort of 9542 breast tumors (5216 from metastatic disease) from patients with advanced breast cancer (Foundation Medicine); 1 of 9542 (0.01%)	late-stage, endocrine-refractory, metastatic (solid tumor; liver metastasis)	comprehensive genomic profiling RNA sequencing	(84)	exon 6	in-frame	unknown	unknown	
ESR1-NKAIN2	cohort of 254 ctDNA samples from patients with advanced breast cancer (Foundation Medicine); 1 of 254 tumors (0.39 %)	Stage IV, endocrine refractory (ctDNA)	comprehensive genomic profiling	(84)	exon 6	in-frame	unknown	unknown	
ESR1-CDK13	cohort of 254 ctDNA samples from patients with advanced breast cancer (Foundation Medicine); 1 of 254 tumors (0.39 %)	Stage IV, endocrine refractory (ctDNA)	comprehensive genomic profiling	(84)	exon 7	in-frame	unknown	unknown	
ESR1-COA5	cohort of 110 advanced ER+ breast cancer patients (Clinical Genotyping Cohort); 1 of 110 (0.9%)	metastatic ER+	anchored multiplex PCR	(71)	exon 4	in-frame	unknown	unknown	
ESR1-PCDH11X	cohort of 25 breast cancers	late stage, endocrine-refractory, metastatic ER+	RNA sequencing	(80)	exon 6	in-frame	stable expression and active fusion protein	estrogen independent growth; fulvestrant resistant growth; constitutive expression of ER and EMT-like transcriptional programs; increased cell motility and development of lung metastasis	(80) (81)
ESR1-NOP2	cohort of 81 primary breast cancers [neoadjuvant AI (NeoAI) Trials]; 1 of 81	primary, treatment-naive, ER+	RNA sequencing	(80)	exon 6	in-frame	stable expression and inactive fusion protein	no role in estrogen independent and ET resistant growth	(80)
ESR1-AKR1D1	cohort of 81 primary breast cancers [neoadjuvant AI (NeoAI) Trials]; 1 of 81	primary, treatment-naive, ER+	RNA sequencing	(80)	exon 6	out-of-frame	N/A	N/A	
ESR1-POLH	cohort of 728 TCGA breast tumors; 1 of 728 (0.13%)	primary	RNA sequencing PCR	(80)	exon 7	in-frame	stable expression and inactive fusion protein	no role in estrogen independent and ET resistant growth	(80)
ESR1-CCDC170	cohort of 728 TCGA breast tumors; 1 of 728 (0.13%)	primary	RNA sequencing	(80)	exon 4	out-of-frame	N/A	N/A	
ESR1-CCDC170	cohort of 728 TCGA breast tumors; 1 of 728 (0.13%)	primary	RNA sequencing	(80)	exon 5	out-of-frame	N/A	N/A	
ESR1-ARNT2-e2	cohort of 2520 pairs of tumor and normal tissues (The Hartwig Medical cohort)	metastatic ER+	whole-genome sequencing	(85)	exon 6	in-frame	stable expression and active fusion protein	estrogen independent growth; ET resistant growth; constitutive expression of ER and EMT-like transcriptional programs; increased cell motility	(81)
ESR1-LPP	cohort of 2520 pairs of tumor and normal tissues (The Hartwig Medical cohort)	metastatic ER+	whole-genome sequencing	(85)	exon 6	in-frame	stable expression and active fusion protein	estrogen independent growth; ET resistant growth; constitutive expression of ER and EMT-like transcriptional programs; increased cell motility	(81)
ESR1-NCOA1	cohort of 2520 pairs of tumor and normal tissues (The Hartwig Medical cohort)	metastatic ER+	whole-genome sequencing	(85)	exon 6	in frame	stable expression and active fusion protein	estrogen independent growth; ET resistant growth; constitutive expression of ER and EMT-like transcriptional programs; increased cell motility	(81)
ESR1-TCF12	cohort of 2520 pairs of tumor and normal tissues (The Hartwig Medical cohort)	metastatic ER+	whole-genome sequencing	(85)	exon 6	in-frame	stable expression and inactive fusion protein	no role in estrogen independent and ET resistant growth	(81)
ESR1-CLINT1	cohort of 2520 pairs of tumor and normal tissues (The Hartwig Medical cohort)	metastatic ER+	whole-genome sequencing	(85)	exon 6	in-frame	stable expression and active fusion protein	estrogen independent growth; ET resistant growth; constitutive expression of ER and EMT-like transcriptional programs; increased cell motility	(81)
ESR1-GRIP1	cohort of 2520 pairs of tumor and normal tissues (The Hartwig Medical cohort)	metastatic ER+	whole-genome sequencing	(85)	exon 6	in-frame	stable expression and active fusion protein	estrogen independent growth; ET resistant growth; constitutive expression of ER and EMT-like transcriptional programs; increased cell motility	(81)
ESR1-TNRC6B	cohort of 2520 pairs of tumor and normal tissues (The Hartwig Medical cohort)	metastatic ER+	whole-genome sequencing	(85)	exon 6	in-frame	stable expression and active fusion protein	estrogen independent growth; ET resistant growth; constitutive expression of ER and EMT-like transcriptional programs; increased cell motility	(81)

Study cohorts, incidence (for patient cohort > n=100), clinical characteristics, identification sites and validation methods are detailed. Functional ESR1 domains preserved in the gene fusion, in frame expression and function of ESR1 fusions are also noted.

## Structure and function of ESR1-e6>fusion proteins in MBC

The ESR1-e2>CCDC170 fusion protein consists of the 5′ untranslated region of ESR1 to the coding region of CCDC170, generating N-terminally truncated CCDC170 proteins (ΔCCDC170) expressed under the *ESR1* promoter (**Figure 1**) (75, 82). Structural studies have revealed that this structure is distinct from the ESR1-e6>fusions identified from ET resistant MBC. Despite the diversity among the ESR1-e6>fusions, they share a common structure whereby the first 6 exons of *ESR1* (ESR1-e6) are preserved, retaining the hormone-independent transactivation domain (TAD) as well as the DNA-binding domain of ER whereas the LBD is lost and replaced with a functional domain of the 3’ fusion partner (**Figure 1**) (76, 81, 84). This structure is strongly associated with estrogen independent growth and ET resistant metastatic ER+ breast tumors. The loss of a functional LBD suggests a clear pathological impact, leading to complete resistance to the activity of current ER antagonists, which all bind to the LBD. As expected, several ESR1-e6>fusions (ESR1-e6>YAP1, ESR1-e6>PCDH11X, ESR1-e6>SOX9 and ESR1-e6>ARNT2-e18) remained stably expressed in the presence of fulvestrant and promoted ET-resistant growth of T-47D and MCF7 cells (80, 81). In contrast, the expression of ER mutant constructs that lack the LBD had decreased transcriptional activity, suggesting that the presence of the 3’ partner is essential for the ER fusion activity (80, 81, 84). The fact that multiple different 3’ partners have the same effect and drive ET resistance and malignant phenotypes, indicates that the enhanced activity of the ER fusions is not dependent on a specific 3’ partners. These findings suggest that the 3’ partner may be important for the stability of ER and possibly the dimerization of ER, however, an intact LBD is not required for ER activity in the context of the ER fusions.

It is important to note that not all ESR1-e6>fusions produce stable proteins with clear transcription factor (TF) or co-activator (CoA) functions, and only a subset of the ESR1-e6>fusions are activating fusions. The number of studies investigating the activity of ESR1-e6>fusions is limited, the function of some fusions are still unknown. Further studies are required to investigate and fully validate the stability and activity of ESR1-e6>fusions. Some ESR1-e6>fusions such as ESR1-e6>YAP1, ESR1-e6>SOX9, ESR1- e6>ARNT2, ESR1-e6>LPP, ESR1-e6>NCOA1, ESR1-e6->PCDH11X, ESR1-e6>CLINT1, ESR1-e6>GRIP1 and ESR1-e6>TNRC6B produce stable and active fusion proteins that are positive regulators of transcription (80, 81). ESR1-e6->DAB2 has cell type specific transcriptional activity- active in MCF7 but not T47D cells. In contrast to transcriptionally active ESR1-e6>fusions, multiple ESR1- e6>fusions (e.g. ESR1-e6>TCF12, ESR1-e6>ARID1B, ESR1-e6>NOP2) were identified as transcriptionally inactive despite producing stable fusion protein, adding to the complex landscape of ESR1- e6>fusion proteins. None of the 3’ partner genes of these latter ESR1-e6>fusions are known to be a TF or CoA and the wild-type protein is not nuclear-localized (80, 81). As hypothesized by Gou and colleagues (81), these transcriptionally inactive ESR1-e6>fusions could (1) encode tumor suppressors, (2) be active exclusively in the presence of a specific set of coactivators, or (3) act as dominant negative regulators interrupting the function of the remaining intact wild-type protein activity.

## Activating ESR1 fusion proteins drive endocrine resistance and metastatic phenotypes

When first described, ESR1-e2>CCDC170 in ER+ breast cancer cells led to enhanced growth and reduced sensitivity to tamoxifen (75) suggesting a role for ESR1-e2>CCDC170 in ET resistance. Additional pre-clinical studies (74, 75, 79) showed that the expression of ESR1-e2>CCDC170 fusions in ER-positive breast cancer cells resulted in increased cell migration, increased colony formation, and increased cell proliferation as evidenced by the increase in the number of cells in S-G2/M phase. Li and colleagues (74) provided detailed evidence supporting the function of ESR1-e2>CCDC170 in promoting breast cancer cell survival and endocrine resistance both *in vitro* and in xenograft models. Their mechanistic study suggests that ESR1-e2>CCDC170 fusions bind and stabilize the HER2/HER3/SRC complex and enhance the activation of SRC/PI3K/AKT signaling during ET *in vitro* and *in vivo*. This study also suggested a potential strategy to manage ESR1-e2>CCDC170 positive patients by combining the HER2 inhibitor lapatinib and/or SRC inhibitor dasatinib with ET.

A series of publications clearly demonstrated that ESR1-e6>fusions were identified from ER+ MBC patients and most ESR1-e6>fusion proteins are drivers of ET resistance (69, 76, 80, 81, 84). Functional properties of these ESR1-e6>fusions include estradiol-independent growth and constitutive expression of ER target genes leading to ET-resistant proliferation and epithelial-mesenchymal transition (EMT) genes facilitating metastasis. The latest and most comprehensive study by Gou and colleagues (81) functionally screened multiple ESR1-e6>fusions and 4 were found to promote estradiol-independent cell growth, migration, EMT and resistance to fulvestrant. The ESR1-e6>YAP1, ESR1-e6>PCDH11X, ESR1-e6>SOX9, and ESR1-e6>ARNT2-e18 fusions promoted cell proliferation and migration in a hormone-independent and fulvestrant-resistant manner in multiple ER+ cell models. Although the four other ESR1-e6>fusions included in this study (ESR1-e6>DAB2, ESR1-e6>GYG1, ESR1-e6>PCMT1, and ESR1-e6>ARID1B) produced stable proteins, they did not promote ET-resistant growth. Moreover, RNA-seq showed that ER-positive breast cancer cells expressing ESR1-e6>YAP1, ESR1-e6>PCDH11X, ESR1-e6>SOX9 and ESR1-e6>ARNT2-e18 fusions upregulated the same cluster of ER target genes that were observed in the control cells stimulated by estradiol and drove constitutive expression of these ER target genes in the absence of estrogen. Pathway analysis also revealed that these transcriptionally active ESR1-e6>fusion proteins upregulated two EMT-related genes, SNAI1 (Snail) and VCAN (versican). The other ESR1-e6>fusions (ESR1-e6>GYG1, ESR1-e6>PCMT1, and ESR1-e6>ARID1B) did not induce estradiol-independent activation of ER and EMT target genes, despite the fact that they translocated to the nucleus. Additional functional studies showed the transcriptionally active ESR1-e6>YAP1 and ESR1-e6>PCDH11X fusions induced cell motility *in vitro* and promoted metastasis to the lung in cell-line xenograft models as well as in a PDX model harboring the ESR1-YAP1 fusion.

## ESR1 fusions as potential biomarkers and novel therapeutic vulnerabilities in breast cancer

Next generation sequencing (NGS) methods such as DNA-based comprehensive whole genome (WGS) or RNA-based transcriptome (WTS) sequencing have been extensively used to describe gene fusions in multiple cancer types (86–90). WGS detects gene fusions based on hybrid-capture methods and still considered the most unbiased approach to identify fusion events, especially in large gene panels. WGS is highly sensitive and can be used on fresh, snap frozen and formalin fixed specimens. Still and all, WGS does not indicate the expression of the gene fusions, and the detection of fusion variants involving large DNA intronic regions is poor (86, 91). WTS have the overall advantage of detecting transcriptionally expressed gene fusions and the sequencing is not affected by intronic regions. Moreover, WTS does not require *a priori* knowledge of gene fusion partners, can distinguish splicing isoforms, quantify fusion transcripts, and it requires low input material. WTS approaches can be based on hybrid-capture or amplicon-based methods using classical or anchor multiplex PCR (86, 92–94). Matissek and colleagues reported anchored multiplex PCR (AMP) as an effective approach to identify gene fusions in cancer, including ER+ metastatic breast cancer (71). AMP was also validated in their study and applied to cohorts of (1) 110 of early-stage and advanced ER+ breast cancer patients (Clinical Genotyping Cohort) and (2) 63 of advanced ER+ breast cancer patients with matched primary and metastatic samples (Matched Primary/Metastasis Cohort). 14 patients in the Clinical Genotyping Cohort harbored intergenic exon–exon fusions, including the in-frame fusion of *ESR1* to *CCDC170*. The authors emphasized that the identified fusion junction sequences involved at least one precise exon boundary. 10 of 63 patients in the Matched Primary/Metastasis Cohort harbored gene fusions in either the primary or metastatic samples. Collectively, AMP detected fusions in 24 of 173 breast cancer patients (14%) in this study, including 11 primary tumors. Combined with complementary “break-apart” fluorescence *in situ* hybridization (FISH) analysis, they further validated AMP as detection technique for clinically relevant fusions. As example, break-apart of *ESR1* was present in primary tumors and metastases from a patient whose tumors demonstrated the ESR1-e4>COA5 fusion upon AMP analysis. Additionally, the ESR1-e2>*CCDC170* fusion detected by AMP was also confirmed upon FISH analysis for *ESR1*. A disadvantage of WTS is that it only identifies expressed fusion genes and not adequate for gene fusion analysis at DNA level. Moreover, biological material is often short, resulting in poor quality RNA and false positive sequencing results. For multiplex PCR approach, the primer design and PCR bias like allele dropout can also impact analysis result (86, 92–94).

ESR1-e6>fusion genes have been detected by NGS methods (**Table 1**), techniques that are not yet employed routinely in the clinic. Analysis of plasma circulating tumor DNA to detect *ESR1* point mutations by droplet digital PCR (ddPCR) is now done in the clinic and clinical trials are investigating the use of these assays in real-time (95–97). Obtaining circulating DNA from liquid biopsies conserves the genomic landscape of the tumor suggesting that this less invasive detection methods may efficiently identify ESR1 fusions, particularly in metastatic ET resistant ER+ breast cancer. Indeed, Hartmaier and colleagues (84) used this approach to provide additional evidence of ESR1 fusion recurrence following extensive ER-targeted endocrine therapies. They obtained target capture sequencing data and examined a cohort of 9542 solid breast tumors and a cohort of 254 ctDNA samples from patients with advanced breast cancer. They successfully identified the ESR1-e6>SOX9, ESR1-e7>MTHFD1L, ESR1-e6>PLEKHG1, and ESR1-e6>TFG fusions (**Table 1**) in four solid tumors and the ESR1-e6>NKAIN2, ESR1-e6>AKAP12, and ESR1-e7>CDK13 (**Table 1**) fusions in 3 ctDNA samples. While there are several commercial platforms available and the cost of ctDNA assay is acceptable in the clinic, the concentration of ctDNA in plasma correlates with tumor size and stage, thus this assay is likely to be useful for late-stage breast cancer patients only (98).

As aforementioned, several ESR1-e6>fusion proteins are inactive and therefore not clinically actionable. As a potential efficient approach for screening samples for the presence of ESR1 fusions that drive ET failure in MBC, Gou and colleagues (81) developed a 24-gene expression signature that is specific for the presence of transcriptionally active ESR1 fusion proteins. Specifically, they identified 24 Hallmark genes, including 19 genes in the estrogen response gene set (CHST8, MAPT, OLFM1, PDZK1, RASGRP1, MPPED2, GREB1, MYB, GFRA1, PGR, ELOVL2, ADCY1, NPY1R, TFF1, ACOX2, SGK1, STC2, CALCR and KRT13), two genes in the EMT gene set (VCAN and COL3A1), and three genes shared in both gene sets (CXCL12, GJA1 and TGM2). To compare the transcriptional profile of ESR1-e6>fusions with known activating *ESR1* LBD point mutations (Y537S and D538G), Gou and colleagues performed RNA-sequencing on T47D cells that overexpressed either several ESR1 fusion proteins (ESR1-e6>ARNT2-e2, ESR1-e6>LPP, ESR1-e6>NCOA1, ESR1-e6>CLINT1, ESR1-e6>TNRC6B and ESR1-e6>GRIP1), or the Y537S and D538G point mutations. ESR1-e6>ARNT2-e2, ESR1-e6>LPP, ESR1-e6>NCOA1, ESR1-e6>CLINT1 and ESR1-e6>TNRC6B demonstrated elevated expression of this gene signature with expression levels comparable to the Y537S and D538G point mutants. Since the LBD point mutants and translocated ESR1 fusions activate a similar pathogenic transcriptional pattern, the gene signature was named “MOTERA” for Mutant or Translocated Estrogen Receptor Alpha. This signature was examined in 20 ER+ patient-derived xenografts and in 55 ER+ MBC samples and successfully identified cases harboring ESR1 fusions.

Gou and colleagues further confirmed the overlap in the transcriptional properties of ESR1-e6>fusions and *ESR1* LBD point mutants in several PDX models and MBC cases. ET-resistant PDXs harboring LBD point mutations (e.g. BCM15100, WHIM20, WHIM40, and HCI013 for ESR1-Y537S; WHIM37 and WHIM43 for ESR1-D538G) highly expressed the MOTERA signature, similar to the PDX naturally expressing the ESR1-e6>YAP1 (WHIM18) fusion. The expression levels of the MOTERA genes were not affected by E2 supplementation in the ESR1-e6>YAP1 expressing PDX or PDXs harboring the LBD point mutations. Furthermore, the MOTERA scores of PDXs expressing WT ESR1 was significantly lower than those of expressing the LBD mutations or the ESR1-e6>YAP1 fusion. Similar to the PDX models, MOTERA gene expression was significantly elevated in MBC tumors harboring the Y537S and D538G point mutations or the ESR1-e6>ARNT2-e18 fusion, and the signature score distinguished the LBD point mutations and the ESR1-e6>ARNT2-e18 fusion from WT *ESR1.*

In addition to the mechanistic studies, evaluating the potential of targeting these fusion proteins for the development of new targeted therapies is critical. Due to the formation and unique structure of ESR1-e6>fusions, all known ET options that target the LBD are ineffective. Lei and colleagues (80) targeted ER signaling regulated by ESR1 fusions by using Palbociclib, a CDK4/6 inhibitor for MBC. ESR1-e6>YAP1 and ESR1-e6>PCDH11X induced cell proliferation was sensitive to a CDK4/6 inhibition, and a PDX naturally expressing the ESR1-e6>YAP1 fusion was also responsive to Palbociclib treatment. Since ESR1 fusion driven growth of ER-positive breast cancers remained sensitive to CDK4/6 inhibition, the presence of an ESR1-e6>fusion could be a putative biomarker to stratify patients for CDK4/6 inhibitor therapy after resistance to endocrine treatment or continued CDK4/6 inhibitor therapy with a second targeted therapy after resistance to first line treatment for metastatic disease with endocrine therapy in combination with a CDK4/6 inhibitor. It is also hypothesized that CDK4/6 inhibition could be beneficial for patients with ESR1 fusions. Further mechanistic and preclinical studies are expected to introduce additional ESR1 fusions sensitive to CDK4/6 inhibitor therapy and expand on the data from patients harboring ESR1 fusions to examine their CDK4/6 inhibitor responses.

To further explore therapeutic strategies that target ESR1 fusions, Gates and colleagues (99) showed that pharmacological inhibition of ESR1-e6>YAP1 fusion with the proteosome inhibitor MG132, blocked ESR1-e6>YAP1 mediated activation of ER target genes. In the same study, bortezomib, a specific 26S proteasome inhibitor, also suppressed growth driven by the ESR1-e6>YAP1 fusion. Bortezomib was tested in a phase II clinical trial in postmenopausal women with ER+ MBC who had progressive disease after prior aromatase inhibitor therapy. The patients were randomized to fulvestrant and bortezomib versus fulvestrant alone groups. In this study, there was no significant difference in progression free survival, which was the primary end point. However, the combination was overall well tolerated and may have enhanced activity in patients who have an ESR1 fusion (100).

## Conclusion and future directions

ET resistance in ER+ breast cancer patients remain a significant clinical problem. The ESR1 fusion proteins are emerging as a mechanism of ET resistance and the studies discussed in this review, deepened our understanding of the prevalence of the ESR1 fusion proteins and the mechanisms by which they drive resistance. The most prevalent and clinically significant ESR1 fusions can be divided into the ESR1-e2>CCDC170 and ESR1-e6>fusion genes. ΔCCDC170, identified in Luminal B breast cancer and generated by ESR1-e2>CCDC170 led to enhanced growth and reduced sensitivity to ET in MBC. ESR1- e6>fusions were identified in ET-resistant MBC and are formed by inter-chromosomal translocation fusing *ESR1* exons 1 to 6 into a 3’ fusion partner, replacing the LBD of ESR1. ESR1-e6>fusions drive estradiol-independent growth and constitutive expression of ER target genes leading to ET-resistance.

ESR1 fusions were identified in more aggressive forms of breast cancer (ET resistant MBC and Luminal B breast cancer) and can guide the diagnosis and the development of therapeutic strategies to treat a subset of patients with tumors that harbor these ESR1 alterations. As for the ESR1-e6>fusion genes, only a handful of functionally active ESR1 fusion proteins have been studied to date and therefore ESR1 fusion events remain an understudied form of somatic mutation in breast cancer. The incidence of these ESR1 fusions is still not well understood, but the studies discussed here collectively suggest that the frequency of ESR1 fusions may be higher in heavily pre-treated metastatic samples and when using more sensitive detection techniques.

The discovery of the *ESR1* LBD point mutations has sparked enthusiasm for the development of a new generation of compounds that not only combat existing ER mutants but also inhibit secondary mutations in ER. Indeed, novel oral SERDs and SERCAs are being developed, and likely to be approved in the clinic. Similar to the emergence of *ESR1* LBD mutations that render ligand independent activity, it is likely that the adoption of more potent SERDs and SERCAs will lead to adaptive mechanisms of resistance that are either ER independent or ER dependent but independent of the LBD. It is currently unclear which mechanisms of resistance may emerge following novel SERD and SERCA treatments.

Although, ESR1 gene fusions are rare, the frequency of these fusions may increase under the selective pressure of more effective SERDs and SERCAs. Therefore, better understanding of the mechanism of action of these fusions that lack the LBD, yet drive tumor progression in ER+ MBC, will be critical for the identification of vulnerabilities to target these fusions.

## Author contributions

All authors listed have made a substantial contribution to the work and approved it for publication.
