# Corrigendum: ESR1 fusions and therapeutic resistance in metastatic breast cancer

**DOI:** 10.3389/fonc.2023.1155540

**Published:** 2023-03-06

**Authors:** Zsuzsanna Nagy, Rinath Jeselsohn

**Affiliations:** ^1^Center for Functional Cancer Epigenetics, Dana Farber Cancer Institute, Harvard Medical School, Boston, MA, United States; ^2^Department of Medical Oncology, Dana-Farber Cancer Institute, Boston, MA, United States; ^3^Department of Medicine, Harvard Medical School, Boston, MA, United States; ^4^Susan F. Smith Center for Women’s Cancers, Dana-Farber Cancer Institute, Harvard Medical School, Boston, MA, United States

**Keywords:** breast cancer, estrogen receptor, ESR1 fusion, endocrine therapy resistance, SERD

In the published article, there was an error. In the main text, some ESR1-e6>fusions that Gou and colleagues characterized were incorrectly referred to as transcriptionally inactive.

A correction has been made to “Structure and function of ESR1- e6>fusion proteins in MBC” section, Paragraph 2. This sentence previously stated:

“ESR1-e6>YAP1, ESR1-e6>SOX9, ESR1- e6>ARNT2, ESR1-e6>LPP, and ESR1-e6>NCOA1 produce active fusion proteins that are positive regulators of transcription (80, 81). In contrast to transcriptionally active ESR1-e6>fusions, multiple ESR1-e6>fusions (ESR1-e6>TCF12, ESR1-e6>ARID1B, ESR1- e6>PCDH11X, ESR1-e6>NOP2, ESR1-e6>DAB2, ESR1- e6>CLINT1, ESR1-e6>GRIP1 and ESR1-e6>TNRC6B) were identified as transcriptionally inactive despite producing stable fusion protein, adding to the complex landscape of ESR1- e6>fusion proteins.”

The corrected sentence appears below:

“The number of studies investigating the activity of ESR1-e6>fusions is limited, the function of some fusions are still unknown. Further studies are required to investigate and fully validate the stability and activity of ESR1-e6>fusions. Some ESR1-e6>fusions such as ESR1-e6>YAP1, ESR1-e6>SOX9, ESR1- e6>ARNT2, ESR1-e6>LPP, ESR1-e6>NCOA1, ESR1-e6->PCDH11X, ESR1-e6>CLINT1, ESR1-e6>GRIP1 and ESR1-e6>TNRC6B produce stable and active fusion proteins that are positive regulators of transcription (80, 81). ESR1-e6->DAB2 has cell type specific transcriptional activity- active in MCF7 but not T47D cells. In contrast to transcriptionally active ESR1-e6>fusions, multiple ESR1-e6>fusions (e.g. ESR1-e6>TCF12, ESR1-e6>ARID1B, ESR1-e6>NOP2) were identified as transcriptionally inactive despite producing stable fusion protein, adding to the complex landscape of ESR1- e6>fusion proteins.”

The authors apologize for this error and state that this does not change the scientific conclusions of the article in any way. The original article has been updated.

